# An Unusual Case of Syringohydromyelia Presenting with Neurogenic Bladder

**DOI:** 10.1055/s-0039-1697925

**Published:** 2019-11-22

**Authors:** Antonella Geljic, Slaven Abdovic, Fran Stampalija, Lana Loncar, Batos A. Tripalo, Martin Cuk

**Affiliations:** 1 Department of Paediatrics, Klinika za djecje bolesti Zagreb, Zagreb, Croatia; 2 Department of Pediatric Nephrology, Klinika za djecje bolesti Zagreb Klinika za pedijatriju, Zagreb, Croatia; 3 Department of Pediatric Urology, Klinika za djecje bolesti Zagreb, Zagreb, Croatia; 4 Department of Pediatric Neurology, Klinika za djecje bolesti Zagreb Klinika za pedijatriju, Zagreb, Croatia; 5 Department of Pediatric Radiology, Klinika za djecje bolesti Zagreb, Zagreb, Croatia

**Keywords:** neurogenic bladder, syringohydromyelia, vesicourethral reflux

## Abstract

We report the case of a 4-year-old boy who first presented with acute pyelonephritis at the age of 6 months. Diagnostic workup revealed high-grade bilateral vesicourethral reflux (VUR). At the age of 18 months, a bulking agent was used to treat bilateral VUR. Since the VUR persisted, an open bilateral Lich-Gregoir procedure was done at the age of 3 years. Immediately after surgery, he developed acute urinary retention with hydronephrosis that resolved with the placement of dwelling urinary catheter. After removal of the catheter urinary retention relapsed so placement of suprapubic urinary catheter was indicated since he did not have sensory loss. He was started with tamsulosin (α − 1-blocker) and prophylactic antibiotics. Urodynamics were performed and suggested bladder outlet obstruction. On the basis of previous urethroscopy and the absence of neurological sequelae, the differential diagnosis of Hinman syndrome was made. After removal of the suprapubic catheter, clean intermittent catheterization was started and α-blocker continued. However, magnetic resonance imaging of the brain and the spinal cord revealed syringohydromyelia extending from thoracic spine (Th5) to conus medullaris with 6 to 7 mm in diameter. Electromyoneurogram was normal. After a follow-up of 3 years, the hydronephrosis has resolved. The patient is on clean intermittent catherization and has no urinary tract infections.

## Introduction

Neurogenic bladder is a dysfunction of the urinary bladder due to disease of the central nervous system or peripheral nerves involved in the control of urination. 1 The most common causes of neurogenic bladder in children are neurospinal dysraphisms such as spina bifida, sacral agenesis, tethered cord, and spinal cord injury. 2 Up to a third of children with neurogenic bladder have vesicoureteral reflux (VUR). 3 In case of neurogenic bladder, the presumed pathomechanism of VUR is a reflux secondary to elevated bladder pressures rather than due to a defective ureterovesical junction. 2 The initial management involves clean intermittent catheterization (CIC) with or without prophylactic antibiotic therapy in combination with an anticholinergic agent. 4 In children with VUR refractory to conservative measures, management includes surgery with ureteral implantation, bladder augmentation, or a combination of treatment methods. 3 Urinary incontinence and bladder dysfunction are rarely described as the first manifestation of syringomyelia, 5 6 a fluid-filled, gliosis-lined cavity within the spinal cord 7 prevalent in 8.4 cases per 100,000 children. 8 Hydromyelia refers to a fluid-filled cavity within the spinal cord lined by ependymal, which likely results from a developmentally nonobliterated central canal. 9 The two terms are often interchanged. The aim of this article was to present an unusual case of syringomyelia that presented with neurogenic bladder without apparent neurological sequels.

## Case Report

**Fig. 1:**
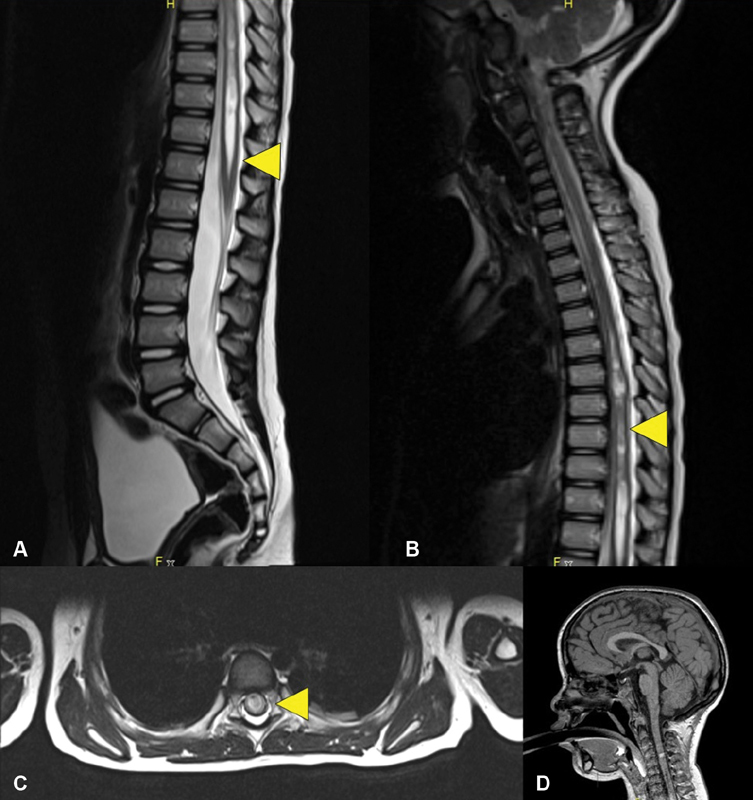
Sagittal T2-weighted magnetic resonance imagings (MRIs) of the patient showing syringohydromyelia from conus medullaris ( **A** ) to thoracic spine, ( **B** ) axial T2-weighted MRI of the syrinx ( **C** ) and cranial MRI without Chiari 1 malformation ( **D** ).

A 1.5-year-old boy was referred to our clinic for the endoscopic treatment of bilateral VUR (grade V on the left side and grade II/III on the right side). He was born after a third pregnancy and was healthy until the age of 6 months when he presented with an episode of acute pyelonephritis. Since spontaneous remission did not occur, instillation of bulking agent bilaterally was done at the age of 18 months. Cystoscopy revealed a bladder with trabeculation. Six months after endoscopic treatment, a contrast-enhanced voiding urosonography was done that revealed still present high-grade VUR on both sides. Since urodynamic study performed at the age of 15 months in other clinic was unremarkable, we chose to perform the Lich-Gregoir procedure with reimplantation of both ureters. Soon after surgery, he developed acute urinary retention with newly established bilateral hydronephrosis. Dwelling urinary catheter was placed after which hydronephrosis resolved. Because of the voiding difficulties and large postvoid residual (PVR), tamsulosin (α-1-adrenergic blocker) and prophylactic antibiotics were initiated. After removal of the catheter urinary retention relapsed so placement of suprapubic urinary catheter was indicated since he did not have sensory loss to initiate CIC. At that point, urodynamic study was repeated in our clinic and bladder outlet obstruction was suggested on the basis of high PQmax of 65 cm H _2_ O. On the basis of unremarkable neurological exam, differential diagnosis was Hinman syndrome. Magnetic resonance imaging (MRI) at this moment was unavailable. Four-hour voiding observation showed significant PVR. After removal of the suprapubic catheter, CIC was started and α-blocker continued. A pediatric neurologist examined the patient and found no abnormal findings; the patient did not have leg weakness or sensory loss. However, MRI of the brain and the spinal cord was done and revealed syringohydromyelia extending from thoracic spine (Th5) to conus medullaris with 6 to 7 mm in diameter ( Fig. 1 ). There were no signs of Chiari 1 malformation on brain scans. Electromyoneurogram (EMNG) of the lower extremities was normal. Neurosurgical consult was done. After a follow-up of 3 years, the hydronephrosis has resolved. The patient is on CIC and has no urinary tract infections. The follow-up ultrasonography demonstrated the right kidney with a size of 8.03 cm, with no hydronephrosis and the hypoplastic left kidney with a size of 5.3 cm.

## Discussion

Most conditions under the group of spinal dysraphism can cause cord tethering and can be associated with syringomyelia. Our case was not associated with Chiari 1 malformation, tethered cord, or any other clear precipitating cause; our patient had idiopathic syringomyelia (IS). There are several studies about the treatment of IS in adult patients, 10 11 but only a few in pediatric population. A two-centered study conducted at Children's Hospital, Boston, Massachusetts and St Luis Children's Hospital, Missouri, concluded that the condition is benign and can be treated conservatively. 12 Another study undertaken at Sheffield Children's Hospital, Sheffield, UK, also concluded that IS is a benign pathology, which can be managed expectantly. 13 Singhal et al also concluded that syringomyelia often remains stable in patients receiving nonoperative treatment. 14 On the basis of first urodynamic study, which was unremarkable, endoscopic antireflux surgery was first treatment of choice for our patient. This procedure has high success rate in primary VUR, while success rates in neurogenic bladder patients have been reported from 53 to 86%. 15 Furthermore, this procedure is less effective in higher grades of reflux and success is generally transient rather than permanent; so patients require long-term follow-up. 16 Since our patient had a persistence of VUR after using the bulking agent, we chose to perform ureteral implantation. At this point, we did not suspect that our patient could have neurogenic bladder since we did not question the validity of first urodynamics. With adequate bladder capacity (% estimated bladder capacity > 70%) and compliance (>7 mL/cm H _2_ O), high grades of reflux have been treated with ureteral implantation alone. 17 18 Postoperative urinary retention after bilateral ureteral reimplantation is common, but is usually transient 19 ; what was not the case with our patient. The differential diagnosis of Hinman syndrome was made on the basis of normal neurological examination and MRI at that moment was not available. It is unusual for syringohydromyelia to present with neurogenic bladder only; it usually presents with back pain, brachial amyotrophy, dissociated sensory loss, and neurogenic arthropathies. 13 Finally, Hinman syndrome, non-neurogenic neurogenic bladder, could still be a differential diagnosis if we consider IS as a benign condition, especially with other neurological sequelae absent and EMNG of the lower extremities normal.

## Conclusion

Neurogenic bladder as the first and only manifestation of syringohydromyelia is rare and can mislead diagnostic workup and delay appropriate therapy. Proper neurologic examination, including MRI, should be done in patients with neurogenic bladder.

## Notes

New Insights and the Importance for the Pediatric Surgeon Syringohydromyelia is a very rare cause of neurogenic bladder in a boy without overt neurological sequels. This is the first reported case in a child to date.
